# Supplementary material for: Splice-Junction-Based Mapping of Alternative Isoforms in the Human Proteome
Source: Cell Rep. Author manuscript; Available in PMC 2020 Jan 15. (PMC6961840; doi:10.1016/j.celrep.2019.11.026)
Supplement: 3 [file NIHMS1546469-supplement-3.zip › DF2/PXD009021/Liver-31-Q9NZN5-LQDGAVVTPSRPLGDTLTVSEAETDPGDVLGR.pdf]

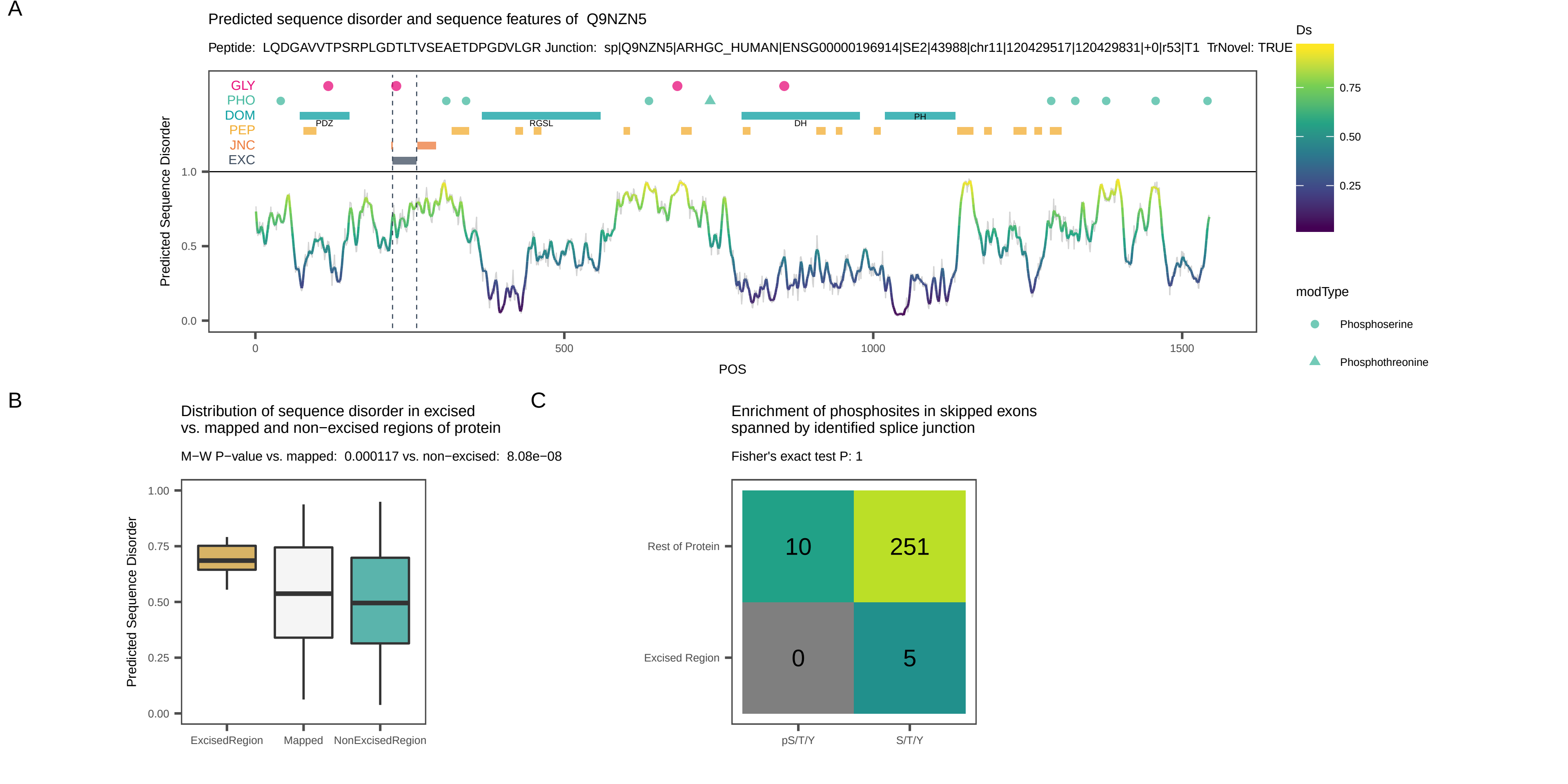
A
Predicted sequence disorder and sequence features of Q9NZN5
Peptide: LQDGAVVTPSRPLGDTLTVSEAETDPGDVLGR Junction: sp|Q9NZN5|ARHGC_HUMAN|ENSG00000196914|SE2|43988|chr11|120429517|120429831|+0|r53|T1 TrNovel: TRUE
GLY
PHO
DOM
PEP
JNC
EXC
PDZ
RGSL
DH
PH
Predicted Sequence Disorder
1.0
0.5
0.0
0
500
1000
1500
POS
Ds
0.75
0.50
0.25
modType
Phosphoserine
Phosphothreonine
B
Distribution of sequence disorder in excised
vs. mapped and non−excised regions of protein
M−W P−value vs. mapped: 0.000117 vs. non−excised: 8.08e−08
Predicted Sequence Disorder
1.00
0.75
0.50
0.25
0.00
ExcisedRegion
Mapped
NonExcisedRegion
C
Enrichment of phosphosites in skipped exons
spanned by identified splice junction
Fisher's exact test P: 1
Rest of Protein
Excised Region
10
251
0
5
pS/T/Y
S/T/Y
